# Longitudinal associations between exclusive, dual and polytobacco use and respiratory illness among youth

**DOI:** 10.21203/rs.3.rs-3793149/v1

**Published:** 2024-01-22

**Authors:** Luis Zavala-Arciniega, Steven Cook, Jana Hirschtick, Yanmei Xie, Richa Mukerjee, Douglas Arenberg, Geoffrey D. Barnes, David T. Levy, Rafael Meza, Nancy Fleischer

**Affiliations:** University of Michigan School of Public Health; University of Michigan School of Public Health; University of Michigan School of Public Health; University of Michigan School of Public Health; University of Michigan School of Public Health; University of Michigan Medical School; University of Michigan Medical School; Georgetown University; BC Cancer Research Institute; University of Michigan School of Public Health

**Keywords:** polytobacco use, respiratory illness, epidemiology

## Abstract

**Background:**

The health consequences of polytobacco use are still well not understand. We evaluated prospective associations between exclusive, dual, and polytobacco use and diagnosed bronchitis, pneumonia, or chronic cough among US youth.

**Methods:**

Data came from Waves 1–5 of the Population Assessment of Tobacco and Health Study. We categorized time-varying past 30-day tobacco use into seven categories: (1) non-current use; exclusive use of 2) cigarettes, 3) electronic nicotine delivery systems (ENDS), or 4) other combustible products (OC; pipes, hookah, and cigars); dual use of 5) ENDS + cigarettes or ENDS + OC 6) cigarettes + OC; or 7) polyuse of all three products. The outcome was incident diagnosis of bronchitis, pneumonia, or chronic cough. We conducted weighted multilevel Poisson models (person n = 17,517, 43,290 observations) to examine the longitudinal exposure-outcome relationship, adjusting for covariates: sex, age, race and ethnicity, parental education, body mass index, secondhand smoke exposure, and household use of combustible products

**Results:**

Compared to nonuse, exclusive cigarette use (Incidence Rate Ratio (IRR) = 1.83, 95% CI 1.25–2.68), exclusive ENDS use (IRR = 1.53, 95% CI 1.08–2.15), combustible product + ENDS dual use (IRR = 1.90, 95% CI 1.18–3.04), cigarettes + OC dual use (IRR = 1.96, 95% CI 1.11–3.48), and polytobacco use (IRR = 3.06 95% CI 1.67–5.63) were associated with a higher incidence of bronchitis, pneumonia, or chronic cough.

**Conclusion:**

We found that exclusive, dual, and poly tobacco use was associated with higher incidence of bronchitis, pneumonia, or chronic cough; Moreover, the incidence rate ratio for polytobacco use was higher than the incidence rate ratio for exclusive use compared to non-current use.

## Introduction

Tobacco use is an important cause of morbidity among youth.^1^ For example, combustible tobacco product use has been identified as a risk factor for acute respiratory diseases.^2,3^ However, little is known about the respiratory health consequences of exclusive, dual (use of two products), and polyuse (three or more products) of tobacco products, which is important due to the continued introduction of new tobacco products.^4,5^ Exclusive, dual, and poly tobacco use patterns are evolving for youth. A recent study using data from 2014 to 2019 found that, among youth, exclusive e-cigarette use increased (from 3.2–12.8%), while exclusive cigarette use and dual/polyuse without e-cigarettes decreased.^6^ Given the rapidly changing tobacco product landscape, it is critical to understand the relationship between exclusive, dual, and polytobacco use and respiratory health outcomes among youth and young adults.

Previous studies have found that cigarette use is associated with increased risk of acute respiratory health outcomes among youth and young adults.^7,8^ For example, clinical and population studies have reported that current cigarette use (vs. never cigarette use) is associated with bronchitis and acute pneumonia among youth and young adults.^7,9–11^ Clinical studies suggested that ENDS use is associated with lipoid pneumonia among young adults.^12–14^ However, population-based studies that have evaluated the association between ENDS use and respiratory diseases among youth have produced mixed results. One longitudinal study in California among high schools students found that current, e-cigarette use (vs. nonuse) was associated with a higher risk of respiratory symptoms.^15^ Results from the Population Assessment of Tobacco and Health (PATH) study reported that current e-cigarette use was not associated with wheezing episodes among youth.^16^ Moreover, results from PATH studies reported that dual use combustible tobacco products was associated with a higher incidence of asthma at follow-up compared to non-use of tobacco products.^17^ In contrast dual use of cigarettes and e-cigarettes cigarettes at baseline was not associated with higher asthma incidence.^17,18^ While asthma association has been prospectively evaluated at national level, there is a need to evaluate the link between exclusive, dual and polytobacco use with short term respiratory outcomes such as acute bronchitis or pneumonia. One recent study of our team found that the exclusive cigarette, exclusive e-cigarette use and dual use of cigarettes and e-cigarettes was associated with bronchitis, pneumonia and chronic cough.^19^ However, no studies have evaluated the link between polytobacco use the risk of bronchitis, pneumonia. We aim to fill this gap by studying this association using data from six waves of the PATH survey, a longitudinal nationally representative study, providing additional insights about the short-term health consequences of polyuse of tobacco products among youth. We hypothesize that the risk of bronchitis, pneumonia or chronic cough will be higher among those who cigarettes, e-cigarettes and other combustible products together.

## Methods

**Figure 1.**
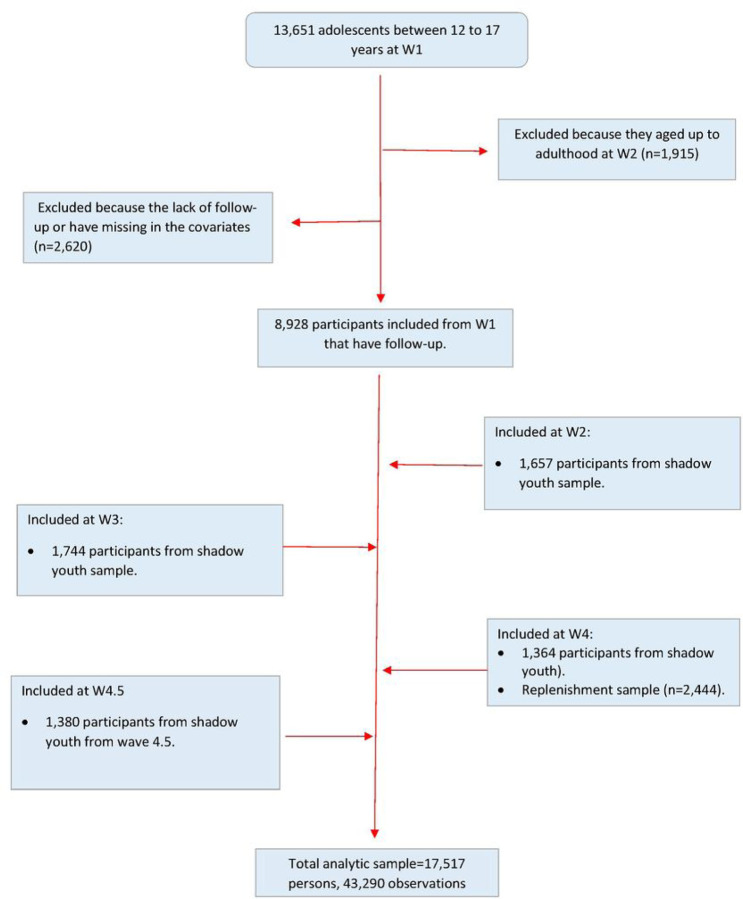
Analytic sample flowchart

We used restricted youth data from waves 1 to 5 of the PATH Study, including wave 4.5. The analytic sample consisted of youth between 12 to 17 years who completed at least one follow-up survey. We also included participants who aged up into the youth sample during W2 to W4.5 (shadow youth) and the replenishment sample in W4. Baseline data for each respondent referred to their first interview, which could occur between W1 to W4.5 (See Fig. 1). Data were collected using audio computer self-interviews (ACASI) in English and Spanish in the following periods: Wave 1 from September 2013 to December 2014; Wave 2 from October 2014 to October 2015; Wave 3 from October 2015 to October 2016; Wave 4 from December 2016 to January 2018; Wave 4.5 from December 2017 to November 2018; and Wave 5 from December 2018 to November 2019. A detailed description of the methodology of the PATH study has been published elsewhere.^20 21^ Given the use of de-identified datasets, the University of Michigan Institutional Review Board deemed this project not regulated as human subject’s research.

### Parent-reported bronchitis, pneumonia, or chronic cough incidence

We evaluated the incidence of parent-reported bronchitis, pneumonia, or chronic cough among youth participants. Parents of the youth participants were queried, *“In the past 12 months, has (Child's first name) been told by a doctor, nurse, or other health professional that (he/she) has bronchitis, pneumonia, or chronic cough?”* The study outcome was measured at each wave, starting in wave 2 and could occur more than once.

### Exclusive, dual, and poly tobacco use as time-dependent exposure variable

For exposure, we categorized time-varying past 30-day tobacco use into 7 mutually exclusive categories: (1) non-current/never tobacco use; exclusive use of (2) cigarettes, (3) electronic nicotine delivery systems (ENDS), and (4) other combustibles (OC; pipes, hookah, and cigars); dual use of (5) ENDS + combustible tobacco (cigarettes or OC), and (6) cigarettes + OC; and (7) polyuse of all three tobacco product groups. The ENDS + combustible tobacco dual use category was created as a combined ENDS + cigarettes and ENDS + OC category due to (a) small sample sizes for ENDS + cigarettes and ENDS + OC, and (b) the hypothesized similar health effect for ENDS plus any combustible product. Current tobacco product use was defined as smoked cigarettes/cigars or use e-cigarettes in the past 30 days. We lagged the exposure variable by one wave (t-1) to ensure that the tobacco variable exposure preceded the bronchitis outcome (i.e., if exposure was measured at W1, the outcome was measured at W2).

### Covariates

We included sex (female, male), race and or ethnicity (Non-Hispanic (NH) White, NH Black, Hispanic, and Another Race/Ethnicity (including multiracial)), parental education (less than high school, high school, some college and bachelor’s degree or higher), body mass index (normal/underweight, overweight, obese), and household use of combustibles products (no, yes) as baseline covariates at the time of respondent’s first interview. Secondhand smoke exposure was measured in number of hours exposed in the past 7 days, cannabis use (yes/no), asthma (yes/no). Age was included as a categorical variable (12–14 and 15–17 years).

### Analysis

First, we created a person-period data set containing multiple responses per participant (n = 17,517; 43,290 observations). We then calculated descriptive statistics for the sociodemographic characteristics and risk factor distributions at baseline for our analytic sample. We also calculated the time-varying prevalence of past 30-day exclusive, dual, and polyuse tobacco exposure variable by wave and the cigarette smoking intensity pattern by wave. Finally, we conducted unadjusted and adjusted weighted, multilevel Poisson models to examine the longitudinal exposure-outcome relationship across five different periods (W1-W2, W2-W3, W3-W4, W4-W4.5, and W4.5-W5). Multilevel models were used because the outcome could occur more than once, and these models adjusted for the lack of independence of the repeated observations. Adjusted models included the covariates described above.

All estimates adjusted for the sample design by recalibrating the PATH weights into two-level weights to accommodate the study longitudinal hierarchy of the data set.^22^ Briefly, Level-1 weights were the conditional wave-specific weights that were scaled, and their sum is equal to the number of data points available for each participant in the study. Level-2 weights were the baseline weights. In other words, Level-2 weights were the cross-sectional weights in which individuals begin in the study (i.e., Wave 1 for most participants, Waves 2–4.5 for aged-up youth, and Wave 4 for youth recruited for the replenishment sample).^22^ We conducted the statistical analysis using Stata 18.1.

## Results

Table 1 shows the baseline sociodemographic characteristics and covariate distribution for participants in our analytic sample at their baseline year (n=17,517). The baseline year corresponds to the wave that the participant entered the study. Just over half the participants were male (51.5%). More than half of respondents (53%) were NH White, 13% were NH Black, 24% were Hispanic, and 10% were from another race/ethnicity. About 40% of participants reported having a parent with a bachelor’s degree or higher. Approximately 27% of the participants reported that someone in their household used tobacco products. A total of 7.4% of the sample reported bronchitis, pneumonia, or chronic cough across the study period. Among respondents who reported the outcome (N=1,309), about 20% (n=264) reported the outcome more than once over the study period. Table 2 describes the changes in the tobacco exposure variable across waves. The prevalence of exclusive use of cigarettes and OC decreased from W1 to W5 (from 1.5% to 0.8%), while exclusive e-cigarette use increased (from 1.1% to 3.8%). Dual use of ENDS with cigarettes or OC did not change from W1 to W5, but cigarette + OC use decreased. Polyuse of tobacco products remained at about 0.3% over the study period. Table 3 shows the cigarette smoking intensity pattern by wave. Cigarette smoking intensity was not statistically different among the exclusive cigarette use, dual use, and polytobacco use categories over time.

**Figure 2.**
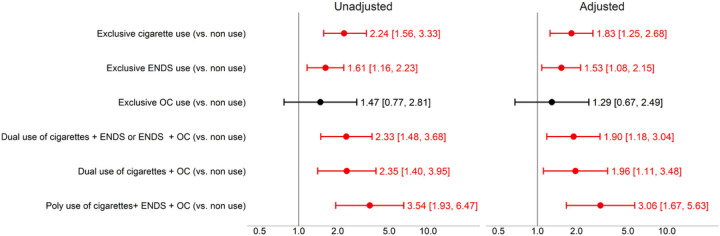
Incidence Rate Ratio of Bronchitis, Pneumonia and Chronic Cough among youth. (Person N=17,517; Observations N=43,290) Models adjusted by age, sex, race and ethnicity, parental education, household use combustible products, asthma, marijuana use, and BMI

The results from the multilevel Poisson regression models can be found in Figure 2 and Supplementary table 1. There were 43,290 observations in the models corresponding to n=17,517 respondents. In the adjusted models, compared to non-current use of tobacco products, exclusive cigarette use (Incidence Rate Ratio [IRR]=1.83, 95% CI 1.25–2.68) and exclusive ENDS use (IRR=1.53, 95% CI 1.08–2.15) were associated with a higher incidence of diagnosed bronchitis, pneumonia, or chronic cough. The risk of diagnosed bronchitis, pneumonia, or chronic cough was also higher for dual use of ENDS + combustible tobacco (IRR=1.90, 95% CI 1.18–3.04), dual use of cigarettes + OC (IRR=1.96, 95% CI 1.11–3.48), and polytobacco use (IRR=3.06 95% CI 1.67–5.63), compared to non-current use of tobacco products. The only tobacco use category that was not statistically different from non-current use was exclusive OC (IRR=1.29, 95% CI 0.67–2.49).

## Discussion

Using data from a large and nationally representative longitudinal sample, we found associations between exclusive, dual, and polyuse of tobacco products and the incidence of acute bronchitis, pneumonia, or chronic cough among youth. Exclusive cigarette use, exclusive ENDS use, dual use of ENDS with combustible tobacco, dual use of cigarettes and OC, and polyuse of cigarettes, ENDS, and OC were all associated with higher risk of acute bronchitis, pneumonia, or chronic cough, with the strongest association for polytobacco use.

The current changing landscape of the tobacco market has led to increased exclusive ENDS use and decreased cigarette use and dual use with cigarettes among youth during the years of our study. However, polytobacco use remained stable during the study period. In this context of availability of multiple tobacco products, we found that adolescents using cigarettes, ENDS, or cigars exclusively or concurrently were at higher risk of developing short-term respiratory outcomes than adolescents who did not use tobacco. Moreover, our findings suggest that polyuse of tobacco products results in greater incidence of bronchitis, pneumonia, or chronic cough. Therefore, policymakers should reinforce measures that restrict access to all tobacco products, including ENDS, for adolescents to reduce their disease risk.

Our finding that exclusive ENDS use and ENDS use with combustible tobacco (dual and poly) were both associated with the incidence of acute bronchitis, pneumonia, or chronic cough suggests that ENDS use among youth affects acute respiratory health. Our finding is generally consistent with findings from clinical studies, which report that e-cigarette use it is related to acute respiratory infections.^23–26^ One potential explanation of the association between exclusive e-cigarette use and acute respiratory infections such as bronchitis and pneumonia is that adolescents frequently share e-cigarette devices with friends and other persons.^27^ Sharing e-cigarette devices potentially could lead to an increase in respiratory infections through the exchange of saliva. Future national surveys on tobacco use should incorporate questions of sharing behaviors of e-cigarettes and other tobacco products.

Consistent with our finding that exclusive cigarette use was associated with acute bronchitis, pneumonia, or chronic cough, previous studies have found that individual combustible tobacco use was associated with bronchitis and pneumonia.^9,10^ By examining dual and polytobacco use, we were further able to demonstrate that the use of two or more tobacco products is associated with bronchitis, pneumonia, and chronic cough. Interestingly, the incidence rate ratio for polytobacco use compared to non-current use was higher than that for exclusive use of any individual product. This association was not explained by differences in cigarette smoking intensity patterns. Of note, cigarette smoking intensity was not statistically different among adolescents who used two or more tobacco products, compared to those who used cigarettes exclusively (Table 3). Therefore, our finding suggests that using two or more tobacco products may further increase the risk of adverse acute respiratory health outcomes relative to exclusive cigarette use.

In contrast, we found that exclusive OC tobacco product use was not statistically associated with an increased risk of incident bronchitis, pneumonia, and chronic cough, but OC use was associated with an increased incident risk when used in combination with other tobacco products. The lack of a statistically significant association of exclusive OC use with the respiratory outcomes could be explained by the smaller sample size for the exclusive OC use category, even though the IRR was above 1. Future research is warranted to examine the independent risk of each product (i.e., hookah, cigars, and pipes) in the OC use category.

This study has several limitations. First, parents were asked about bronchitis, chronic cough, and pneumonia as part of a single question in the PATH survey, so that it was not possible to separately examine these respiratory outcomes. Future longitudinal studies incorporating specific questions for each respiratory health outcome are warranted. Second, diagnosis of the respiratory outcomes were self-reported by the parents and not confirmed clinically, which might introduce information bias. Third, we cannot rule out residual confounding in our analyses. For example, PATH study assessed urbanicity of residence only at W1, so we were unable to adjust for it given the inclusion of youth shadow samples (i.e., youth who began participating in the study in W2 or after). Fourth, the outcome survey question was only assessed for youth but not adults, limiting our ability to follow participants when they aged up into the adult sample. Despite these limitations, to our knowledge, this study is important because provide new evidence of the relationship between specific tobacco products used alone or in combination and acute respiratory health effects among youth.

## Conclusion

In the context of the rapidly changing tobacco use patterns among youth, we found that, compared to non-current use, the exclusive use of cigarettes, the exclusive use of ENDS, and the use of two or more tobacco products were strongly associated with incident bronchitis, pneumonia, and chronic cough. Policymakers should carefully consider reinforcing measures that restrict youth access to all tobacco products, including ENDS, to protect the health of the youth population in the U.S.

## Supplementary Material

Supplement 1

## Figures and Tables

**Table 1.** Baseline sociodemographic characteristics and smoking behavior, Population Assessment of Tobacco & Health Study (Wave 1, 2013, 2014) among youth (n=17,517)

	%	95% CI	n
**Sex**			
Male	51.5	[50.7, 52.2]	9098
Female	48.5	[47.7, 49.3]	8416
**Age** *			
12–14 years	60.5	[59.4,61.6]	5437
15–17 years	39.5	[38.4,40.6]	3525
**Race/ethnicity**			
NH White	53.0	[40.2,53.8]	8390
NH Black	12.9	[12.4,13.5]	2280
Hispanic	24.1	[23.4,24.7]	5160
Another Race/Ethnicity	10.0	[9.5,10.5]	1678
**Parental education**			
Less than high school	13.0	[12.5,13.5]	2686
High school	20.7	[20.1,21.4]	3853
Some college	27.0	[26.2,27.7]	4730
Bachelor's degree or higher	39.2	[38.5,40.5]	6248
***Baseline risk factors***			
**Second hand smoke exposure (10 hours)**	0.3	[0.3,0.3]	17,517
**Ever marijuana use**			
No	**93.6**	[92.2,94.0]	16289
Yes	**6.4**	[6.0,8.8]	1228
**Ever asthma diagnosis**			
No	**81.9**	[81.3,82.5]	14288
Yes	**18.1**	[17.5,18.7]	3229
**Household use of combustible products**			
No	73.5	[72.7,74.1]	12667
Yes	26.5	[25.9,27.3]	4850
**BMI – obesity**			
Normal/underweight	64.8	[64,0,65.4]	11064
Overweight	18.3	[17.7,18.9]	3290
Obese	17.0	[16.3,17.6]	3163
**Bronchitis, Pneumonia or Chronic cough episodes** **			
None	92.6	[92.2,93.0]	16208
One	5.9	[5.5,6.2]	1045
Two or more	1.5	[1.3,1.7]	264

* Table 1 only includes the distribution of age at W1. Age was included as time-variant in the models

** Bronchitis, pneumonia and Chronic cough episodes over the study period. From W2 to W5

**Table 2.** Time-Dependent tobacco exposure variable by wave (n=43,290 observations)

	Wave 1 (n=8928)		
*Time varying poly tobacco use variable*	%	95% CI	n
Non use	95.1	[94.6, 95.5]	8485
Exclusive cigarette use	1.5	[1.2, 1.8]	132
Exclusive e-cigarette use	1.1	[0.9, 1.3]	91
Exclusive other combustible use	0.7	[0.5, 0.9]	66
Dual use of cigarettes + ENDS/ Dual use of ENDS + OC	0.7	[0.6, 0.9]	64
Dual use of cigarettes + other combustibles	0.5	[0.4, 0.7]	48
Poly use of cigarettes+ ENDS + OC	0.4	[0.3, 0.6]	43
	**Wave 2 (n=8,271)**		
Non use	95.0	[94.5, 95.5]	7855
Exclusive cigarette use	1.3	[1.1, 1.6]	113
Exclusive e-cigarette use	1.4	[1.2, 1.7]	114
Exclusive other combustible use	0.8	[0.6, 1.0]	68
Dual use of cigarettes + ENDS/ Dual use of ENDS + OC	0.7	[0.5, 0.9]	59
Dual use of cigarettes + other combustibles	0.5	[0.3, 0.6]	40
Poly use of cigarettes+ ENDS + OC	0.3	[0.2, 0.4]	22
	**Wave 3 (n=7,825)**		
Non use	95.6	[95.1, 96.1]	7469
Exclusive cigarette use	0.9	[0.7, 1.2]	70
Exclusive e-cigarette use	1.9	[1.6, 2.2]	151
Exclusive other combustible use	0.4	[0.3, 0.6]	38
Dual use of cigarettes + ENDS/ Dual use of ENDS + OC	0.6	[0.5, 0.9]	54
Dual use of cigarettes + other combustibles	0.3	[0.2, 0.5]	27
Poly use of cigarettes+ ENDS + OC	0.2	[0.1, 0.3]	16
	**Wave 4 (n=9,614)**		
Non use	95.6	[95.1, 96.0]	9192
Exclusive cigarette use	1.0	[0.8, 1.2]	99
Exclusive e-cigarette use	2.0	[1.7, 2.4]	178
Exclusive other combustible use	0.4	[0.3, 0.6]	41
Dual use of cigarettes + ENDS/ Dual use of ENDS + OC	0.6	[0.5, 0.8]	64
Dual use of cigarettes + other combustibles	0.2	[0.1, 0.3]	19
Poly use of cigarettes+ ENDS + OC	0.2	[0.1, 0.3]	21
	**Wave 4.5 (n=8,652)**		
	%	95% CI	n
Non use	93.8	[93.3, 94.4]	8108
Exclusive cigarette use	0.8	[0.6, 1.0]	75
Exclusive e-cigarette use	3.8	[3.4, 4.3]	328
Exclusive other combustible use	0.3	[0.2, 0.4]	26
Dual use of cigarettes + ENDS/ Dual use of ENDS + OC	0.8	[0.6, 1.0]	72
Dual use of cigarettes + other combustibles	0.2	[0.1, 0.3]	18
Poly use of cigarettes+ ENDS + OC	0.3	[0.2, 0.4]	25

**Table 3.** Time-Dependent smoking intensity variable (cigarettes smoked per day=CPD) by wave

Wave 1			
	Mean cigarettes per day (CPD)	95% CI	n
Exclusive cigarette use	1.73	[1.15, 2.32]	132
Dual use of cigarettes + ENDS or ENDS+ OC	1.85	[0.87, 2.82]	64
Dual use of cigarettes + OC	1.62	[0.87, 2.38]	48
Polyuse of cigarettes + ENDS + OC	2.38	[1.12, 3.64]	43
Wave 2			
Exclusive cigarette use	1.86	[1.04, 2.69]	111
Dual use of cigarettes + ENDS or ENDS+ OC	1.62	[0.76, 2.49]	59
Dual use of cigarettes + OC	3.71	[2.30, 5.12]	40
Polyuse of cigarettes + ENDS + OC	0.96	[0.30, 1.62]	22
Wave 3			
Exclusive cigarette use	1.61	[0.86, 2.36]	70
Dual use of cigarettes + ENDS or ENDS+ OC	2.19	[0.84, 3.54]	54
Dual use of cigarettes + OC	2.49	[1.12, 3.86]	27
Polyuse of cigarettes + ENDS + OC	2.43	[0.88, 3.99]	16
Wave 4			
Exclusive cigarette use	1.20	[0.68, 1.72]	99
Dual use of cigarettes + ENDS or ENDS+ OC	0.52	[0.21, 0.82]	64
Dual use of cigarettes + OC	2.68	[0.08, 5.28]	19
Polyuse of cigarettes + ENDS + OC	1.32	[0.69, 1.95]	21
Wave 4.5			
Exclusive cigarette use	1.11	[0.53, 1.69]	75
Dual use of cigarettes + ENDS or ENDS+ OC	0.74	[0.20, 1.29]	72
Dual use of cigarettes + OC	3.30	[0.83, 5.76]	19
Polyuse of cigarettes + ENDS + OC	1.60	[0.24, 2.96]	25
