# Supplementary material for: Longitudinal associations between exclusive, dual and polytobacco use and respiratory illness among youth
Source: Res Sq. 2024 Jan 22:rs.3.rs-3793149. Preprint. [Version 1] doi: 10.21203/rs.3.rs-3793149/v1 (PMC10854317; doi:10.21203/rs.3.rs-3793149/v1)
Supplement: Supplement 1 [file NIHPPRS3793149v1-supplement-1.pdf]

Supplementary Files

This is a list of supplementary files associated with this preprint. Click to download.

- SupplementaryTable1.12.21.23.docx
